# Dual improvement of alopecia areata and immune thrombocytopenia with baricitinib: a case report

**DOI:** 10.1093/skinhd/vzae019

**Published:** 2025-01-22

**Authors:** Chelsea Moon, Sarah E Park, Jennifer L Hsiao, Katrina H Lee

**Affiliations:** Keck School of Medicine, University of Southern California, Los Angeles, CA, USA; David Geffen School of Medicine, University of California Los Angeles, Los Angeles, CA, USA; Department of Dermatology, Keck School of Medicine, University of Southern California, Los Angeles, CA, USA; Department of Dermatology, Keck School of Medicine, University of Southern California, Los Angeles, CA, USA

## Abstract

The oral Janus kinase (JAK) inhibitor baricitinib is approved by the U.S. Food and Drug Administration for the treatment of alopecia areata (AA). We report a case of dual improvement of AA and immune thrombocytopenia (ITP) with oral baricitinib monotherapy, which may suggest linked autoimmune pathophysiology. In phase III clinical trials of baricitinib for AA, reports of rare adverse haematological events include neutropenia and anaemia. While a history of haematological comorbidities may raise concern for many clinicians when considering treatment with a JAK inhibitor, this clinical vignette suggests that baricitinib may be considered and safely administered in those with concomitant AA and ITP. A 56-year-old man with a history of AA, ITP and vitiligo presented to the clinic for relapse of his steroid-resistant hair loss which had previously been treated with tofacitinib. In consultation with the patient’s haematologist, baricitinib 2 mg daily was started with close platelet monitoring then doubled to 4 mg after platelets showed improvement at the 6-month follow-up. Fourteen months after initiating baricitinib, improvement in white and dark hair regrowth was observed, and platelets remained normal. Thus, baricitinib may be considered for the dual treatment of AA and ITP with regular platelet monitoring and co-management with haematology colleagues.

## Teaser

A 56-year-old male with history of AA, ITP and vitiligo presented to clinic for relapse of his steroid-resistant hair loss which was previously treated with tofacitinib. After initiating baricitinib monotherapy, sustained improvement in hair growth and platelet counts were observed. While history of haematologic comorbidities may raise concern for many clinicians when considering treatment with a JAK inhibitor, this clinical vignette suggests that baricitinib may be considered and safely administered in those with concomitant AA and ITP.

**What is already known about this topic?**

- Baricitinib therapy is approved by the U.S. Food and Drug Administration to treat alopecia areata (AA) and preliminary trials have suggested its potential efficacy in the treatment of immune thrombocytopenia (ITP).

**What does this study add?**

- Our patient had a rare presentation of concurrent AA and ITP.
- Baricitinib may be considered for dual treatment of AA and ITP with regular platelet monitoring and co-management with haematology despite reports of serious haematological adverse effects with other Janus kinase inhibitors.

##  

Alopecia areata (AA) is an autoimmune-mediated, non-­scarring hair loss disorder that results from lymphocytic attack of the hair bulb.^1^ Traditional treatments for AA include intralesional and topical corticosteroids, systemic corticosteroids, topical immunotherapy and other immunosuppressants.^2^ Baricitinib, an oral Janus kinase (JAK) inhibitor, was approved by the U.S. Food and Drug Administration (FDA) in 2022 for the treatment of severe AA in adults.^1^ AA has well-documented associations with a variety of autoimmune disorders, such as vitiligo, autoimmune thyroid disease and lupus erythematosus.^1^ A limited number of cases of concurrent AA and immune thrombocytopenia (ITP), an immune-mediated platelet disorder, have also been described in the literature.^3–6^ Herein, we report a case of improved AA and ITP after the initiation of baricitinib.

## Case report

**Figure 1:**
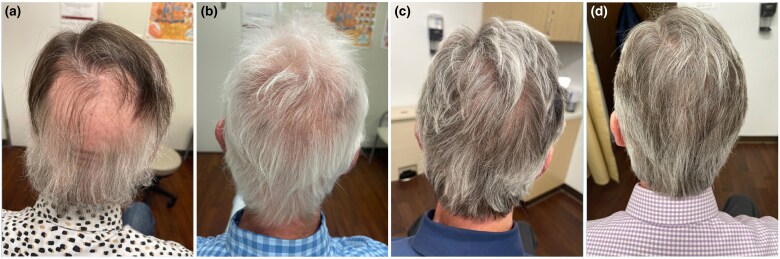
Before and during baricitinib treatment. (a) Nonscarring alopecia of the vertex and occipital scalp at baseline. (b) Regrowth of the patient’s with poliosis on baricitinib 2 mg daily at the 6-month follow-up. (c) Regrowth with hair repigmentation at the 12-month follow-up. (d) Full regrowth of hair over the vertex and occipital scalp with improved repigmentation 14 months after initiating baricitinib monotherapy.

A 56-year-old man with history of AA, ITP and vitiligo presented to the clinic for management of his hair loss. His AA was refractory to 3 months of intralesional steroid injections but responded to a 3-month course of 10 mg oral tofacitinib with robust hair growth. Oral tofacitinib therapy was subsequently tapered over 3 months and the patient’s AA remained in remission for 2 years. A few months prior to presentation, the patient reported relapse of his AA. Physical examination revealed broad patches of nonscarring hair loss of the scalp over the vertex, occipital and parietal regions with an approximate Severity of Alopecia Tool (SALT) score of 50 (Figure 1a). Baseline laboratory values included white blood cell count 4800 cells μL^–1^, haemoglobin 13.3 g dL^–1^ and a decreased platelet count of 82 000 cells μL^–1^. Two years before relapse of his hair loss, the patient had been hospitalized for their ITP and recovered after a 6-month course of prednisolone. Quantiferon Gold, and hepatitis B and C serologies were non reactive.

**Figure 2:**
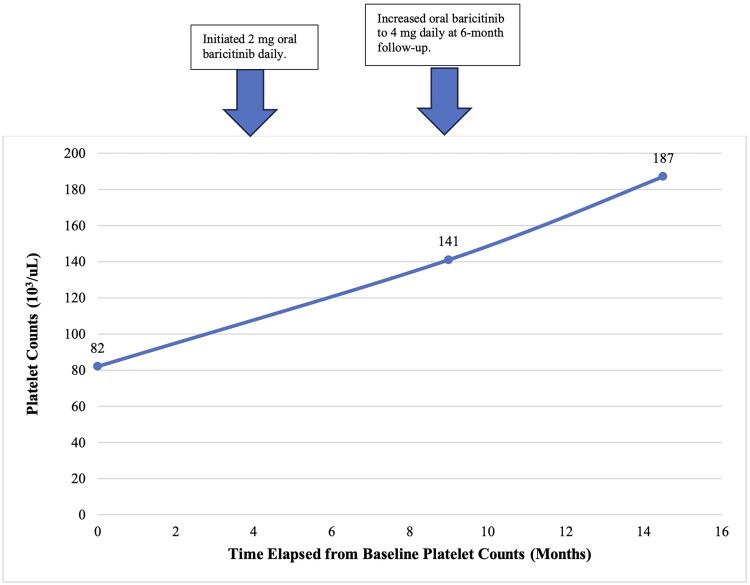
Platelet levels before and during oral baricitinib treatment for alopecia areata.

In consultation with the patient’s haematologist, baricitinib 2 mg daily was started with close platelet monitoring. At the 6-month follow-up, the patient had regrowth of hair on the scalp, eyebrows, legs, arms and hands (Figure 1b). The platelet count had improved to 141 000 μL^–1^ (Figure 2). Baricitinib dose was subsequently increased to 4 mg daily. Six months later, increased hair density with a mix of dark and white hair growth was observed (Figure 1c), and the platelet count entered normal range at 187 000 μL^–1^ (Figure 2). At 14 months, marked improvement in white and dark hair regrowth was observed (Figure 1d), and the patient’s count remained within the normal range. Haemoglobin levels remained within the normal range during the course of oral baricitinib therapy. The patient did not develop anaemia or neutropenia while on baricitinib.

## Discussion

Oral and topical JAK inhibitors have been approved by the FDA for various inflammatory diseases of the skin, such as psoriasis and AA.^1^ Current JAK inhibitors approved for the treatment of AA include baricitinib and ritlecitinib.^1^

There are currently two reports of simultaneous AA and ITP in paediatric patients, and two reports of co-­occurring AA and ITP in an adult.^3–6^ An adult patient with AA and ITP showed improvement in both conditions with oral corticosteroids and intravenous immunoglobulin.^6^ To our knowledge, this is the first case report to demonstrate improvement of both AA and ITP with oral baricitinib ­monotherapy.

In a normal hair follicle, downregulation of major histocompatibility complex (MHC) class I expression prevents autoantigen presentation to CD8^+^ cytotoxic T cells and confers immune privilege to the hair follicle.^7^ AA pathophysiology represents the breakdown of immune privilege. In AA, the lymphocytic infiltrate of the hair bulb carries CD8^+^ cytotoxic T cells with elevated interleukin-15 and interferon (IFN)-γ, which are maintained by a positive feedback loop.^7^ IFN-γ activates the JAK 1/2 pathway along the T helper 1 (Th1) axis to upregulate MHC class I expression, rendering hair follicles vulnerable to autoimmune attack.^1^ Thus, the inhibition of the JAK/STAT pathway has been a proposed therapeutic target in clinical and laboratory settings. In murine models of AA, baricitinib administration reduced CD8^+^ lymphocyte infiltration and MHC class I expression, and IFN-γ returned to normal levels, presumably recovering hair follicle immune privilege.^8^

In the phase III BRAVE-AA1 and BRAVE-AA2 clinical trials, baricitinib 2 and 4 mg daily were found to be superior to ­placebo when evaluated by SALT score in patients with AA.^2^ Common side-effects of baricitinib include upper respiratory infection, acne and nausea.^2^ Rare serious haematological adverse effects observed in BRAVE-AA1 and BRAVE-AA2 included neutropenia and anaemia, which may raise concerns for providers whose patients with AA have haematological ­comorbidities.^2^

ITP is a diagnosis of exclusion in patients with a platelet count below 100 000 μL^–1^, where other aetiologies, such as infection and autoimmune disorders, have been ruled out.^9^ Much about the pathogenesis of ITP has yet to be uncovered, but it is postulated that autoantigens formed from phagocytosed platelets are presented by MHC class II molecules to facilitate autoreactive Th cell activation and autoantibody production against platelets.^9^ ITP has been characterized by a pathogenic predominance of Th1 and Th17 cells, and a decline in regulatory T-cell presence.^9^ Similar to the role cytotoxic T cells play in AA, Th1 cell involvement in ITP could suggest increased IFN-γ release and successive activation of the JAK/STAT pathway.

Platelet normalization in this patient may be explained by JAK 1/2 inhibition tempering Th1 cell autoreactivity in a mechanism similar to the recovery of hair follicle immune privilege in AA. In murine models of JAK2 deletion in platelets and megakaryocytes (Mk), thrombocytosis via reduced thrombopoietin turnover and expansion of Mk-biased haematopoietic stem cell lineages has been observed and may offer an additional explanation for platelet recovery in this patient.^10^

For patients with haematological abnormalities who are on a JAK inhibitor, regular monitoring of blood counts and co-management with haematology colleagues should be considered. In the literature, there is one report of ITP remission achieved by oral JAK inhibition in a patient with ulcerative colitis.^11^ The safety and efficacy of baricitinib for the treatment of steroid-resistant or relapsed ITP was assessed in an open-­label, single-center phase II clinical trial, which found that 20 of 35 patients in the oral baricitinib 4 mg daily group achieved a durable 6-month response.^12^ There are currently two ongoing clinical trials assessing the efficacy and safety of baricitinib for treating steroid-resistant ITP (NCT#05852847 and 05932524). Our case suggests that baricitinib may be a beneficial treatment option for patients with AA who also have ITP. Further large-scale studies are needed to elucidate the safety, efficacy and role of baricitinib for the management of ITP.

## Data Availability

Data sharing is not applicable to this article as no new data were created or analysed in this study.
